# Supplementary material for: Respiratory symptoms after coalmine fire and pandemic: A longitudinal analysis of the Hazelwood Health Study adult cohort
Source: PLOS Glob Public Health. 2025 Jan 22;5(1):e0004186. doi: 10.1371/journal.pgph.0004186 (PMC11753711; doi:10.1371/journal.pgph.0004186)
Supplement: S1 Text — (DOCX) [file pgph.0004186.s001.docx]

Supplementary materials: Long-term effects of coalmine fire-related PM_2.5_ on respiratory symptoms: a longitudinal analysis of the Hazelwood Health Study adult cohort

Table of Contents

[1 Survey items 3](#_Toc186793345)

[1.1 Respiratory symptom questions 3](#_Toc186793346)

[1.2 Self-reported COVID-19 4](#_Toc186793347)

[2 Model specifications 5](#_Toc186793348)

[3 Descriptives 7](#_Toc186793349)

[3.1 Differences between cohort members based on follow-up participation 8](#_Toc186793350)

[3.2 Changes in respiratory symptoms between surveys 10](#_Toc186793351)

[4 Model output tables 11](#_Toc186793352)

[5 Sensitivity analyses 13](#_Toc186793353)

[6 Deviations from pre-registered analysis plan 15](#_Toc186793354)

[7 Additional resources 16](#_Toc186793355)

[7.1 Statistical packages for cleaning and analysing study data 16](#_Toc186793356)

[7.2 Mapping data and software 16](#_Toc186793357)

[8 References 17](#_Toc186793358)

# Survey items

## Respiratory symptom questions

Respiratory symptom items were derived the *European Community Respiratory Health Survey III – Screening Questionnaire* [1] and are reproduced here.

- **Chronic cough:** Do you cough on most days for as much as three months a year?
- **Current wheeze:** Have you had wheezing or whistling in your chest at any time in the last 12 months?
- **Chest tightness:** Have you woken up with a feeling of tightness in your chest at any time in the last 12 months?
- **Shortness of breath (nocturnal):** Have you been woken by an attack of shortness of breath at any time in the last 12 months?
- **Shortness of breath (resting):** Have you had an attack of shortness of breath that came on during the day when you were at rest at any time in the last 12 months?
- **Current nasal symptoms:** Have you had a problem with sneezing or a runny nose when you did not have a cold or the flu **in the last 12 months**??
- **Chronic phlegm:** Do you bring up phlegm from your chest on most days for as much as three months a year?

Wet chronic cough was determined by presence of both chronic cough and chronic phlegm, whereas dry chronic cough was determined by presence of chronic cough and *absence* of chronic phlegm.

## Self-reported COVID-19

To determine whether a participant had ever had self-reported COVID-19, the survey included the following item from the *Avon Longitudinal Study of Parents and Children* [2]. Any of the yes answers were classified as having a history COVID-19.

- Do you think that you currently have or have had COVID-19?

1. Yes, confirmed by a positive test
2. Yes, suspected by a doctor but not tested
3. Yes, my own suspicions
4. No (Go to …)

# Model specifications

We constructed Directed Acyclic Graphs (DAGs) using the *DAGitty* [3] software to determine which covariates to include in our analytical models, based on our theoretical understanding of causal relationships. Node colours correspond to the type of variable. We opted for the minimal sufficiently-adjusted model in each case. For the longitudinal fire-related PM_2.5_ analysis, minimal confounder adjustment consisted of demographics (age and sex), socioeconomic status (IRSAD score and educational attainment), and study site (exposure site Morwell or control site Sale). For fire-related PM_2.5_*COVID-19 analysis, this was demographics, socioeconomic status, study site, occupational exposure, smoker status/pack-years, and pre-fire asthma or COPD.


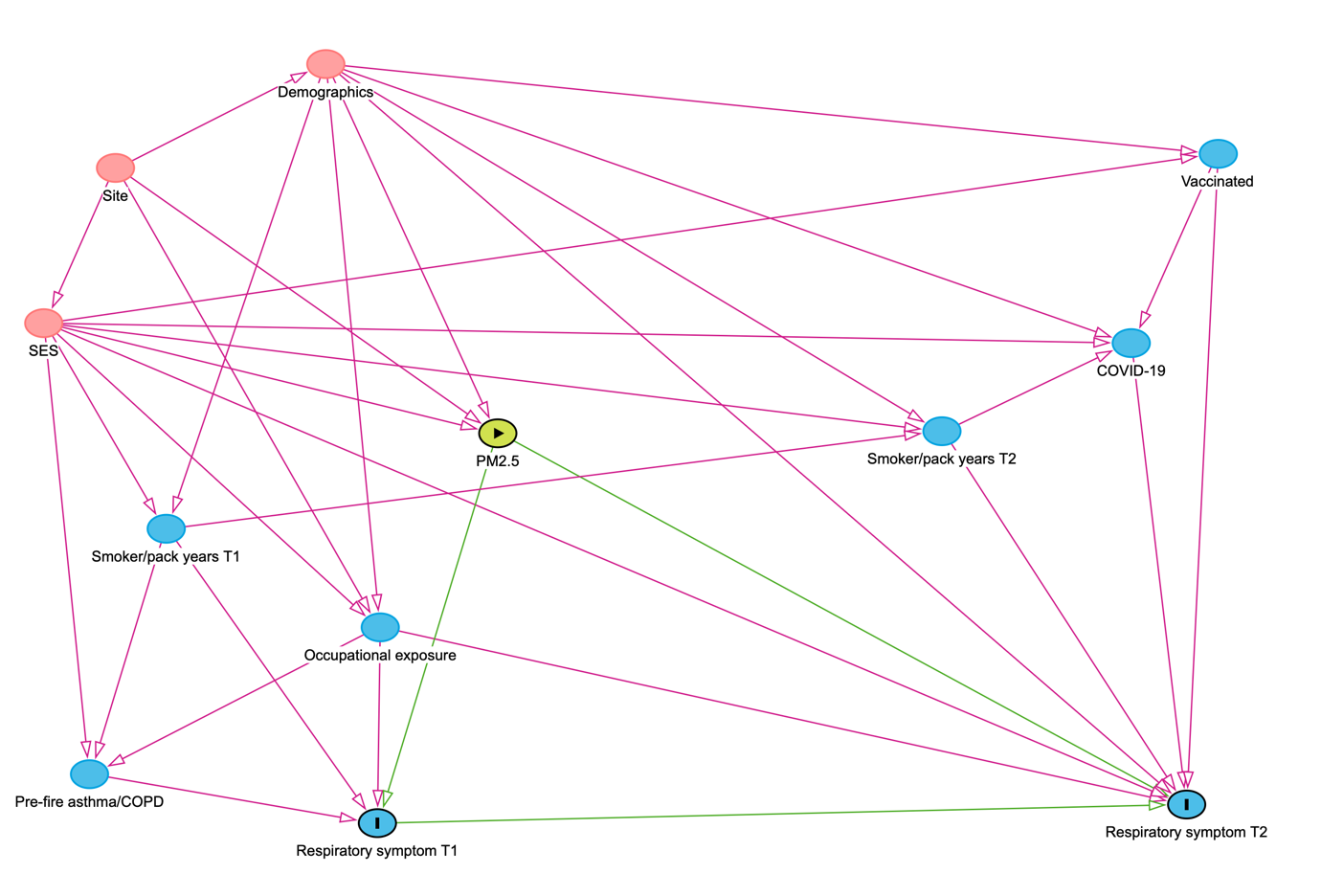


Figure A. Directed Acyclic Graph (DAG) to determine what variables were necessary and appropriate to adjust for in our model evaluating the longitudinal effects of fire-related PM_2.5_; green nodes with an arrow are exposures, blue nodes with an “I” are outcomes, red nodes are adjusted confounders, and blue nodes without an “I” are outcome exposure antecedents that either should *not* be adjusted or are unnecessary to adjust for


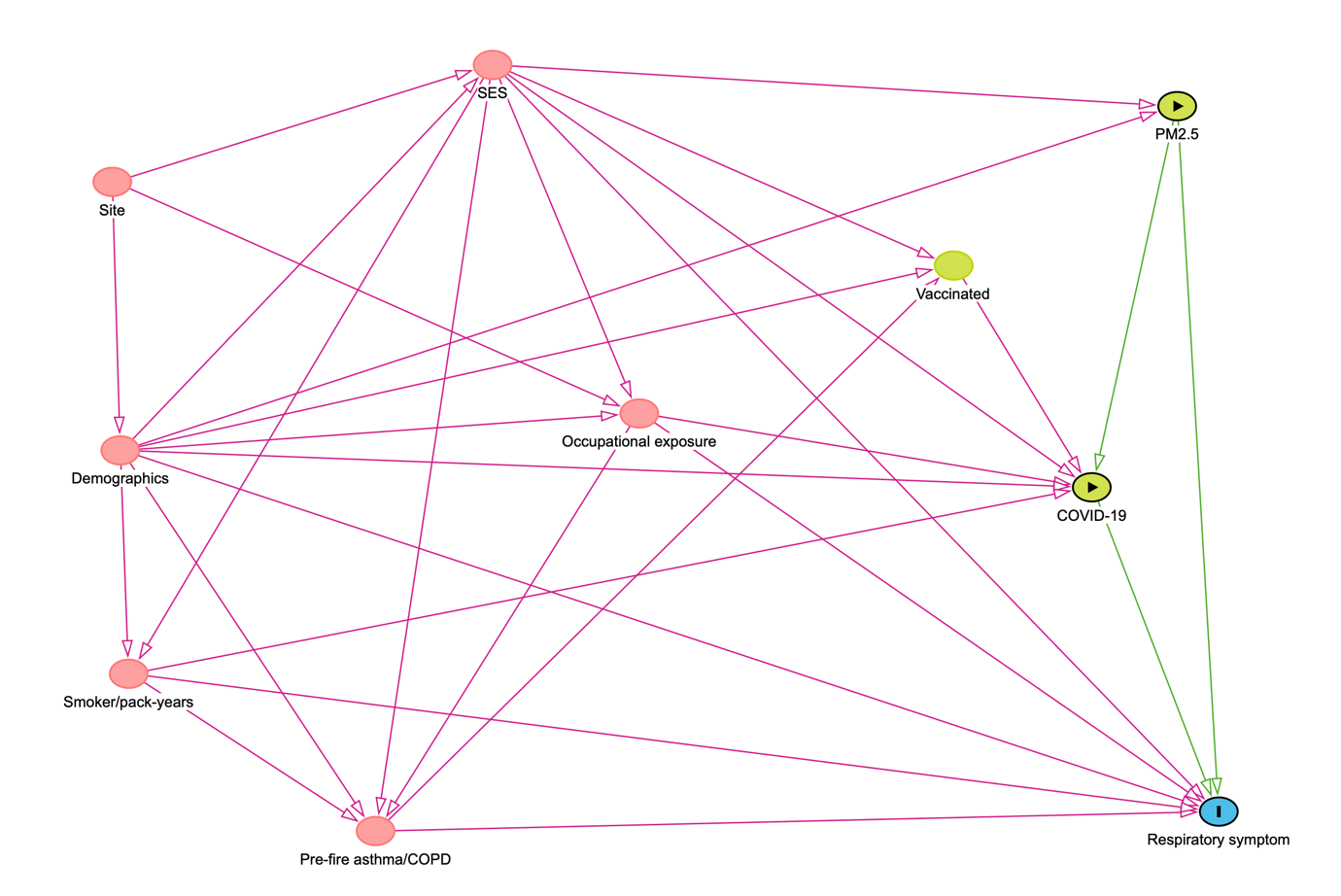


Figure B. Directed Acyclic Graph (DAG) to determine what variables were necessary and appropriate to adjust for in our model evaluating the interacting effects of fire-related PM_2.5_ and COVID-19 on respiratory symptoms; green nodes with an arrow are exposures, blue nodes with an “I” are outcomes, red nodes are adjusted confounders

# Descriptives

Table A. Sample descriptive statistics, divided by residence during the mine fire (Morwell or Sale) and survey round (2016-2017 original or 2022 follow-up); count variables are presented as *n* (%) and continuous as median (IQR)

|  | **2016-2017 original survey** | | **2022 follow-up survey** | |
| --- | --- | --- | --- | --- |
|  | **Morwell (*n* = 3096)** | **Sale (*n* = 960)** | **Morwell (*n* = 408)** | **Sale (*n* = 204)** |
| Mean 24-hour PM_2.5_ exposure µg/m^3^ | 11 [IQR: 7, 19] | 0 [IQR: 0, 0] | 11 [IQR: 8, 19] | 0 [IQR: 0, 0] |
| Ever had COVID-19  *Missing* | - | - | 168 (41%)  *3* | 86 (42%)  *0* |
| Full COVID-19 vaccination (4+)  *Missing* | - | - | 238 (59%)  *3* | 104 (51%)  *1* |
| **Demographics/confounders** | | | | |
| Age at survey  *Missing* | 59 [IQR: 46, 70]  *5* | 59 [IQR: 45, 70]  *0* | 63 [IQR: 54, 71]  *1* | 64 [IQR: 55, 73]  *0* |
| Female | 1705 (55%) | 550 (57%) | 236 (58%) | 126 (62%) |
| Educational attainment  Secondary up to year 10  Secondary, years 11-12  Certificate/diploma/tertiary  *Missing* | 1006 (33%)  668 (22%)  1381 (45%)  *41* | 241 (25%)  162 (17%)  546 (58%)  *11* | 98 (24%)  80 (20%)  228 (56%)  *2* | 29 (14%)  41 (20%)  133 (66%)  *1* |
| Occupational exposure  Coalmine/power station  Other exposure  Not exposed | 494 (16%)  777 (25%)  1825 (59%) | 29 (3.0%)  323 (34%)  608 (63%) | 71 (17%)  106 (26%)  231 (57%) | 7 (3.4%)  50 (25%)  147 (72%) |
| Smoker status  Non-smoker  Previous smoker  Current smoker  *Missing* | 1495 (49%)  1052 (34%)  516 (17%)  *33* | 498 (52%)  325 (34%)  126 (13%)  *11* | 190 (47%)  167 (41%)  47 (12%)  *4* | 107 (53%)  84 (42%)  11 (5.4%)  *2* |
| Cigarette pack-years (excl. non-smokers) | 14 [IQR: 5, 30] | 15 [IQR: 5, 26] | 12 [IQR: 4, 24] | 20 [IQR: 5, 30] |
| Socioeconomic score (IRSAD) [4]  *Missing* | 833 [IQR: 782, 889]  *75* | 898 [IQR: 844, 952]  *0* | 838 [IQR: 782, 909]  *14* | 930 [IQR: 844,952]  *0* |
| Asthma (pre-2014)  *Missing* | 715 (23%)  *17* | 205 (21%)  *5* | 116 (29%)  *1* | 44 (22%)  *0* |
| COPD (pre-2014)  *Missing* | 148 (4.8%)  *10* | 27 (2.8%)  *4* | 11 (2.7%)  *2* | 2 (1.0%)  *0* |
| **Outcomes** | | | | |
| Chronic cough  *Missing* | 989 (32%)  *21* | 189 (20%)  *6* | 170 (42%)  *4* | 44 (22%)  *0* |
| Chronic cough (wet)  *Missing* | 600 (20%)  *28* | 107 (11%)  *6* | 92 (23%)  *6* | 24 (12%)  *0* |
| Chronic cough (dry)  *Missing* | 385 (13%)  *28* | 82 (8.6%)  *6* | 77 (19%)  *6* | 29 (9.8%)  *0* |
| Current wheeze  *Missing* | 1317 (43%)  *16* | 254 (27%)  *5* | 207 (51%)  *3* | 67 (33%)  *0* |
| Chest tightness  *Missing* | 792 (26%)  *16* | 154 (16%)  *3* | 132 (33%)  *3* | 42 (21%)  *0* |
| Shortness of breath (nocturnal)  *Missing* | 635 (21%)  *15* | 120 (13%)  *3* | 108 (27%)  *3* | 25 (12%)  *1* |
| Shortness of breath (resting)  *Missing* | 611 (20%)  *27* | 90 (9.4%)  *3* | 112 (28%)  *1* | 32 (16%)  *0* |
| Current nasal symptoms  *Missing* | 1,358 (44%)  *8* | 330 (34%)  *3* | 241 (59%)  *2* | 100 (49%)  *0* |
| Chronic phlegm  *Missing* | 785 (26%)  *21* | 150 (16%)  *3* | 115 (28%)  *2* | 31 (15%)  *0* |

## Differences between cohort members based on follow-up participation

There were numerous differences between members of the cohort who took part in the 2022 follow-up survey and those who did not. These are listed in Table S2. Much of this was likely related to the decrease in the proportion of socioeconomically-deprived Morwell residents taking part, from 78% in the 2016/17 original survey to 67% in the follow-up. Follow-up participants were more likely to be younger at the original survey, have completed a higher level of education, be a former smoker and not a current smoker, and live in a higher socioeconomic area. Participants were more likely to have had pre-fire asthma but less likely to have had pre-fire COPD. Notably, there were few detectable differences in respiratory symptom outcomes. These included a higher prevalence of current nasal symptoms and lower prevalence chronic phlegm.

Table B. Differences in key variables between cohort members based on participation in the follow-up survey

|  | **Follow-up (*n* = 612)** | **Not in follow up (*n* = 3,444)** | ***p-value*** |
| --- | --- | --- | --- |
| Morwell (versus Sale) | **408 (67%)** | **2,688 (78%)** | **<0.001** |
| Mean 24-hour PM_2.5_ exposure µg/m^3^ | **8 [IQR: 0, 14]** | **9 [IQR: 3, 14]** | **<0.001** |
| **Demographics/confounders** | | | |
| Age at survey  *Missing* | **58 (49, 65)**  ***1*** | **60 (45, 71)**  ***5*** | **0.002** |
| Female | 362 (59%) | 1,893 (55%) | 0.058 |
| Educational attainment  Secondary up to year 10  Secondary, years 11-12  Certificate/diploma/tertiary  *Missing* | **127 (21%)**  **121 (20%)**  **361 (59%)**  ***3*** | **1,120 (33%)**  **709 (21%)**  **1,566 (46%)**  ***49*** | **<0.001** |
| Occupational exposure  Coalmine/power station  Other exposure  Not exposed  *Missing* | **78 (13%)**  **156 (25%)**  **378 (62%)** |  |  |
| Smoker status  Non-smoker  Previous smoker  Current smoker  *Missing* | **286 (47%)**  **249 (41%)**  **74 (12%)**  ***3*** | **1,707 (50%)**  **1,128 (33%)**  **568 (17%)**  ***41*** | **<0.001** |
| Cigarette pack-years (excl. non-smokers) | 13 [IQR: 5, 25]  *296* | 15 [IQR: 5, 30]  *1,815* | 0.199 |
| Socioeconomic score (IRSAD) [4]  *Missing* | **869 [IQR: 814, 936]**  ***14*** | **839 [IQR: 800, 909]**  ***61*** | **<0.001** |
| Asthma (pre-2014)  *Missing* | **160 (26%)**  ***1*** | **760 (22%)**  ***21*** | **0.032** |
| COPD (pre-2014)  *Missing* | **13 (2.1%)**  ***2*** | **162 (4.7%)**  ***12*** | **0.002** |
| **Outcomes** | | | |
| Chronic cough  *Missing* | 182 (30%)  *3* | 996 (29%)  *24* | 0.699 |
| Chronic cough (wet)  *Missing* | 100 (16%)  *4* | 607 (18%)  *30* | 0.452 |
| Chronic cough (dry)  *Missing* | 81 (13%)  *4* | 386 (11%)  *30* | 0.169 |
| Current wheeze  *Missing* | 241 (39%)  *0* | 1,330 (39%)  *18* | 0.822 |
| Chest tightness  *Missing* | 148 (24%)  *2* | 798 (23%)  *17* | 0.604 |
| Shortness of breath (nocturnal)  *Missing* | 131 (21%)  *0* | 624 (18%)  *18* | 0.063 |
| Shortness of breath (resting)  *Missing* | 117 (19%)  *4* | 584 (17%)  *26* | 0.202 |
| Current nasal symptoms  *Missing* | **295 (48%)**  ***1*** | **1,393 (41%)**  ***10*** | **<0.001** |
| Chronic phlegm  *Missing* | **118 (19%)**  ***3*** | **817 (24%)**  ***221*** | **0.016** |

## Changes in respiratory symptoms between surveys

Using a crude mixed-effects logistic regression, we compared the prevalence of respiratory symptoms between the initial and follow-up surveys between study sites. While the prevalence of each symptom increased in Morwell, in Sale there were increases in four of the nine symptoms: current wheeze, chest tightness, resting shortness of breath, and nasal allergy. These effects are summarised in Figure S1.


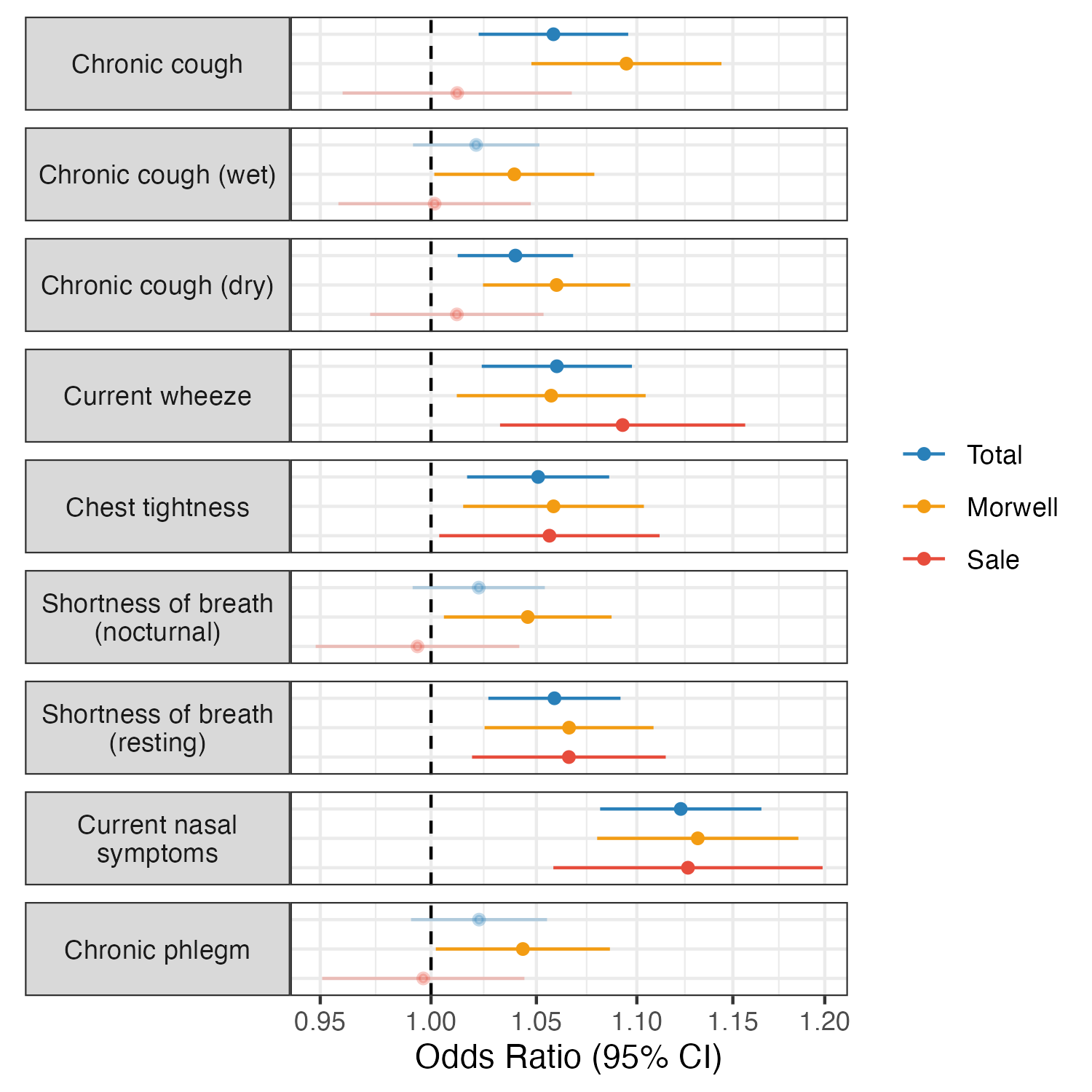


Figure C. Changes in the prevalence of respiratory symptoms between the initial 2016/17 survey (3-4 years post-fire) and the 2022 follow-up (8.5-9 years post-fire)

# Model output tables

Table C. Effects of 10µg/m^3^ increase in mean coalmine fire-related PM_2.5_ on odds of respiratory symptoms at the initial 2016/17 survey and changes to the 2022 follow-up; significant associations at *p* ≤ 0.05 in bold

| **Respiratory symptom** | **Model type** | **PM_2.5_ effect at initial survey** | **Change in PM_2.5_ effect since initial survey** | **Long-term PM_2.5_ effect** |
| --- | --- | --- | --- | --- |
| **Chronic cough** | Crude | **1.23 (1.15-1.31, p = 0.000)** | **1.24 (1.05-1.47, p = 0.012)** | **1.52 (1.30-1.79, p = 0.000)** |
|  | Adjusted | **1.16 (1.08-1.24, p = 0.000)** | **1.22 (1.03-1.45, p = 0.022)** | **1.41 (1.20-1.67, p = 0.000)** |
|  | Adjusted - adding site | **1.09 (1.01-1.18, p = 0.022)** | **1.20 (1.02-1.43, p = 0.033)** | **1.31 (1.11-1.56, p = 0.001)** |
| **Chronic cough (wet)** | Crude | 1.21 (0.93-1.56, p = 0.150) | 1.29 (0.87-1.92, p = 0.213) | **1.55 (1.03-2.35, p = 0.037)** |
|  | Adjusted | 1.13 (0.86-1.49, p = 0.378) | 1.28 (0.85-1.92, p = 0.231) | 1.45 (0.94-2.24, p = 0.092) |
|  | Adjusted - adding site | 1.09 (0.79-1.49, p = 0.610) | 1.28 (0.85-1.93, p = 0.239) | 1.39 (0.87-2.20, p = 0.164) |
| **Chronic cough (dry)** | Crude | 1.12 (0.84-1.50, p = 0.453) | 1.06 (0.70-1.60, p = 0.786) | 1.18 (0.76-1.85, p = 0.454) |
|  | Adjusted | 1.10 (0.81-1.50, p = 0.550) | 1.06 (0.70-1.60, p = 0.797) | 1.16 (0.73-1.84, p = 0.525) |
|  | Adjusted - adding site | 1.03 (0.72-1.47, p = 0.883) | 1.05 (0.69-1.61, p = 0.824) | 1.08 (0.65-1.78, p = 0.769) |
| **Current wheeze** | Crude | **1.20 (1.13-1.27, p = 0.000)** | **1.19 (1.01-1.41, p = 0.033)** | **1.43 (1.23-1.67, p = 0.000)** |
|  | Adjusted | **1.14 (1.07-1.21, p = 0.000)** | 1.17 (1.00-1.38, p = 0.057) | **1.33 (1.14-1.56, p = 0.000)** |
|  | Adjusted - adding site | 1.04 (0.97-1.12, p = 0.287) | 1.15 (0.97-1.35, p = 0.098) | **1.19 (1.02-1.40, p = 0.031)** |
| **Chest tightness** | Crude | **1.17 (1.08-1.26, p = 0.000)** | 1.04 (0.87-1.25, p = 0.680) | **1.21 (1.02-1.44, p = 0.032)** |
|  | Adjusted | **1.12 (1.03-1.22, p = 0.006)** | 1.02 (0.85-1.22, p = 0.850) | 1.14 (0.95-1.37, p = 0.151) |
|  | Adjusted - adding site | 1.04 (0.96-1.14, p = 0.348) | 1.00 (0.83-1.21, p = 0.971) | 1.05 (0.87-1.26, p = 0.631) |
| **Shortness of breath (nocturnal)** | Crude | 1.14 (0.89-1.45, p = 0.297) | 1.30 (0.87-1.93, p = 0.201) | 1.48 (0.97-2.25, p = 0.069) |
|  | Adjusted | 1.09 (0.84-1.41, p = 0.521) | 1.29 (0.86-1.94, p = 0.213) | 1.41 (0.91-2.18, p = 0.124) |
|  | Adjusted - adding site | 1.01 (0.75-1.37, p = 0.933) | 1.29 (0.86-1.95, p = 0.219) | 1.31 (0.82-2.08, p = 0.252) |
| **Shortness of breath (resting)** | Crude | 1.22 (0.96-1.55, p = 0.112) | 0.97 (0.66-1.42, p = 0.866) | 1.18 (0.79-1.74, p = 0.417) |
|  | Adjusted | 1.16 (0.89-1.51, p = 0.264) | 0.95 (0.64-1.40, p = 0.786) | 1.10 (0.73-1.66, p = 0.653) |
|  | Adjusted - adding site | 1.05 (0.77-1.42, p = 0.765) | 0.93 (0.62-1.39, p = 0.721) | 0.97 (0.62-1.53, p = 0.906) |
| **Current nasal symptoms** | Crude | **1.13 (1.07-1.20, p = 0.000)** | 0.93 (0.80-1.08, p = 0.343) | 1.05 (0.91-1.21, p = 0.514) |
|  | Adjusted | **1.11 (1.04-1.18, p = 0.001)** | 0.92 (0.79-1.08, p = 0.315) | 1.02 (0.88-1.19, p = 0.750) |
|  | Adjusted - adding site | 1.05 (0.98-1.12, p = 0.204) | 0.91 (0.78-1.06, p = 0.242) | 0.95 (0.82-1.11, p = 0.534) |
| **Chronic phlegm** | Crude | 1.24 (0.99-1.57, p = 0.064) | 1.10 (0.75-1.60, p = 0.625) | 1.37 (0.92-2.02, p = 0.117) |
|  | Adjusted | 1.15 (0.90-1.48, p = 0.262) | 1.08 (0.73-1.59, p = 0.695) | 1.25 (0.83-1.87, p = 0.292) |
|  | Adjusted - adding site | 1.10 (0.82-1.46, p = 0.527) | 1.07 (0.73-1.58, p = 0.729) | 1.17 (0.76-1.82, p = 0.474) |

Table D. Effects of 10µg/m^3^ increases in coalmine fire-related PM_2.5_ and COVID-19 on prevalence of respiratory symptoms and whether there was a moderating effect, 8.5-9 years after the fire; faded points and intervals indicate non-significant effects; significant associations at *p* ≤ 0.05 in bold

| **Respiratory symptom** | **Model type** | **PM_2.5_ effect at initial survey** | **Change in PM_2.5_ effect since initial survey** | **Long-term PM_2.5_ effect** |
| --- | --- | --- | --- | --- |
| **Chronic cough** | Crude | **1.42 (1.18-1.70, p = 0.000)** | 1.07 (0.81-1.41, p = 0.652) | **1.51 (1.22-1.86, p = 0.000)** |
|  | Adjusted | **1.39 (1.13-1.71, p = 0.002)** | 1.00 (0.74-1.36, p = 0.977) | **1.39 (1.11-1.75, p = 0.005)** |
|  | Adjusted - adding site | **1.31 (1.04-1.64, p = 0.022)** | 1.01 (0.75-1.36, p = 0.945) | **1.32 (1.03-1.69, p = 0.026)** |
| **Chronic cough (wet)** | Crude | **1.23 (1.01-1.50, p = 0.039)** | 1.34 (0.99-1.81, p = 0.054) | **1.65 (1.32-2.07, p = 0.000)** |
|  | Adjusted | 1.21 (0.96-1.52, p = 0.104) | 1.29 (0.93-1.79, p = 0.122) | **1.56 (1.22-1.99, p = 0.000)** |
|  | Adjusted - adding site | 1.17 (0.91-1.51, p = 0.222) | 1.30 (0.94-1.81, p = 0.115) | **1.52 (1.18-1.97, p = 0.001)** |
| **Chronic cough (dry)** | Crude | **1.31 (1.07-1.61, p = 0.009)** | 0.79 (0.56-1.10, p = 0.166) | 1.03 (0.79-1.36, p = 0.813) |
|  | Adjusted | **1.27 (1.01-1.59, p = 0.040)** | 0.77 (0.54-1.10, p = 0.148) | 0.97 (0.72-1.30, p = 0.847) |
|  | Adjusted - adding site | 1.18 (0.92-1.52, p = 0.188) | 0.76 (0.52-1.10, p = 0.144) | 0.89 (0.64-1.25, p = 0.509) |
| **Current wheeze** | Crude | 1.27 (1.06-1.53, p = 0.008) | 1.25 (0.93-1.67, p = 0.135) | **1.59 (1.27-2.00, p = 0.000)** |
|  | Adjusted | 1.19 (0.96-1.46, p = 0.105) | 1.30 (0.95-1.78, p = 0.105) | **1.54 (1.20-1.98, p = 0.001)** |
|  | Adjusted - adding site | 1.16 (0.92-1.46, p = 0.217) | 1.30 (0.95-1.77, p = 0.106) | **1.50 (1.14-1.96, p = 0.003)** |
| **Chest tightness** | Crude | 1.09 (0.90-1.32, p = 0.368) | 1.13 (0.86-1.49, p = 0.384) | **1.23 (1.01-1.51, p = 0.042)** |
|  | Adjusted | 1.01 (0.81-1.25, p = 0.931) | 1.20 (0.89-1.62, p = 0.223) | 1.22 (0.97-1.52, p = 0.087) |
|  | Adjusted - adding site | 0.92 (0.72-1.18, p = 0.520) | 1.22 (0.90-1.66, p = 0.195) | 1.13 (0.89-1.44, p = 0.322) |
| **Shortness of breath (nocturnal)** | Crude | 1.15 (0.94-1.41, p = 0.169) | 1.21 (0.90-1.61, p = 0.203) | **1.39 (1.13-1.72, p = 0.002)** |
|  | Adjusted | 1.06 (0.85-1.31, p = 0.631) | 1.26 (0.93-1.71, p = 0.138) | **1.33 (1.06-1.67, p = 0.014)** |
|  | Adjusted - adding site | 0.93 (0.72-1.20, p = 0.570) | 1.29 (0.94-1.78, p = 0.115) | 1.20 (0.94-1.53, p = 0.144) |
| **Shortness of breath (resting)** | Crude | **1.31 (1.09-1.58, p = 0.005)** | 0.86 (0.65-1.14, p = 0.298) | 1.13 (0.91-1.40, p = 0.272) |
|  | Adjusted | **1.26 (1.02-1.56, p = 0.031)** | 0.85 (0.62-1.15, p = 0.291) | 1.07 (0.84-1.36, p = 0.580) |
|  | Adjusted - adding site | 1.20 (0.95-1.51, p = 0.126) | 0.85 (0.62-1.16, p = 0.298) | 1.02 (0.78-1.32, p = 0.904) |
| **Current nasal symptoms** | Crude | 1.02 (0.85-1.21, p = 0.845) | 1.07 (0.82-1.40, p = 0.606) | 1.09 (0.89-1.33, p = 0.393) |
|  | Adjusted | 1.00 (0.83-1.20, p = 0.995) | 1.06 (0.81-1.39, p = 0.657) | 1.06 (0.86-1.31, p = 0.573) |
|  | Adjusted - adding site | 0.91 (0.74-1.12, p = 0.356) | 1.07 (0.82-1.40, p = 0.627) | 0.97 (0.77-1.21, p = 0.782) |
| **Chronic phlegm** | Crude | 1.18 (0.98-1.43, p = 0.079) | 1.33 (1.00-1.77, p = 0.054) | **1.57 (1.27-1.95, p = 0.000)** |
|  | Adjusted | 1.15 (0.92-1.43, p = 0.211) | 1.28 (0.94-1.75, p = 0.114) | **1.47 (1.17-1.86, p = 0.001)** |
|  | Adjusted - adding site | 1.09 (0.85-1.39, p = 0.494) | 1.30 (0.95-1.77, p = 0.104) | **1.41 (1.10-1.81, p = 0.007)** |

# Sensitivity analyses

Longitudinal analyses of respiratory symptoms suggested coalmine fire-related PM_2.5_ exposure increased the prevalence of current wheeze. Earlier Hazelwood Health Study analyses found limited evidence that Morwell residents had poorer asthma control, though there was no detectable association with PM_2.5_. The authors postulated that poorer asthma control in Morwell could nevertheless be explained by asthma being exacerbated by smoke exposure [5].

Using self-reported asthma at the initial 2016/17 survey, we conducted stratified longitudinal analyses of PM_2.5_ exposure’s effect on respiratory symptoms. The results suggest effects were mostly isolated to non-asthmatics, including increased prevalence of chronic cough, current wheeze, chest tightness, and chronic phlegm. There was also a reduction in chest tightness among asthmatics, which could be due to increased use in asthma medications.

The asthmatic subsample had less statistical power due to smaller numbers (*n* = 920 at the initial 2016/17 survey and 160 at the 2022 follow-up, compared to *n* = 3,114 and *n* = 451 among non-asthmatics). While this could mean that effects were harder to detect within asthmatics, point estimates indicate the exacerbation of respiratory symptoms was isolated to non-asthmatics. As we note in the *Discussion* of the main document, this may be because asthmatics have access to inhaled medications like corticosteroids that could control respiratory symptoms brought on by the coalmine fire. For instance, majority of asthmatics (*n* = 601, 60.2%) reported taking some sort of medication, though we have no further details about kind or frequency. Medication access may also explain why chest tightness in decreased in asthmatics between the initial and follow-up surveys. These findings are illustrated in Figure S4.


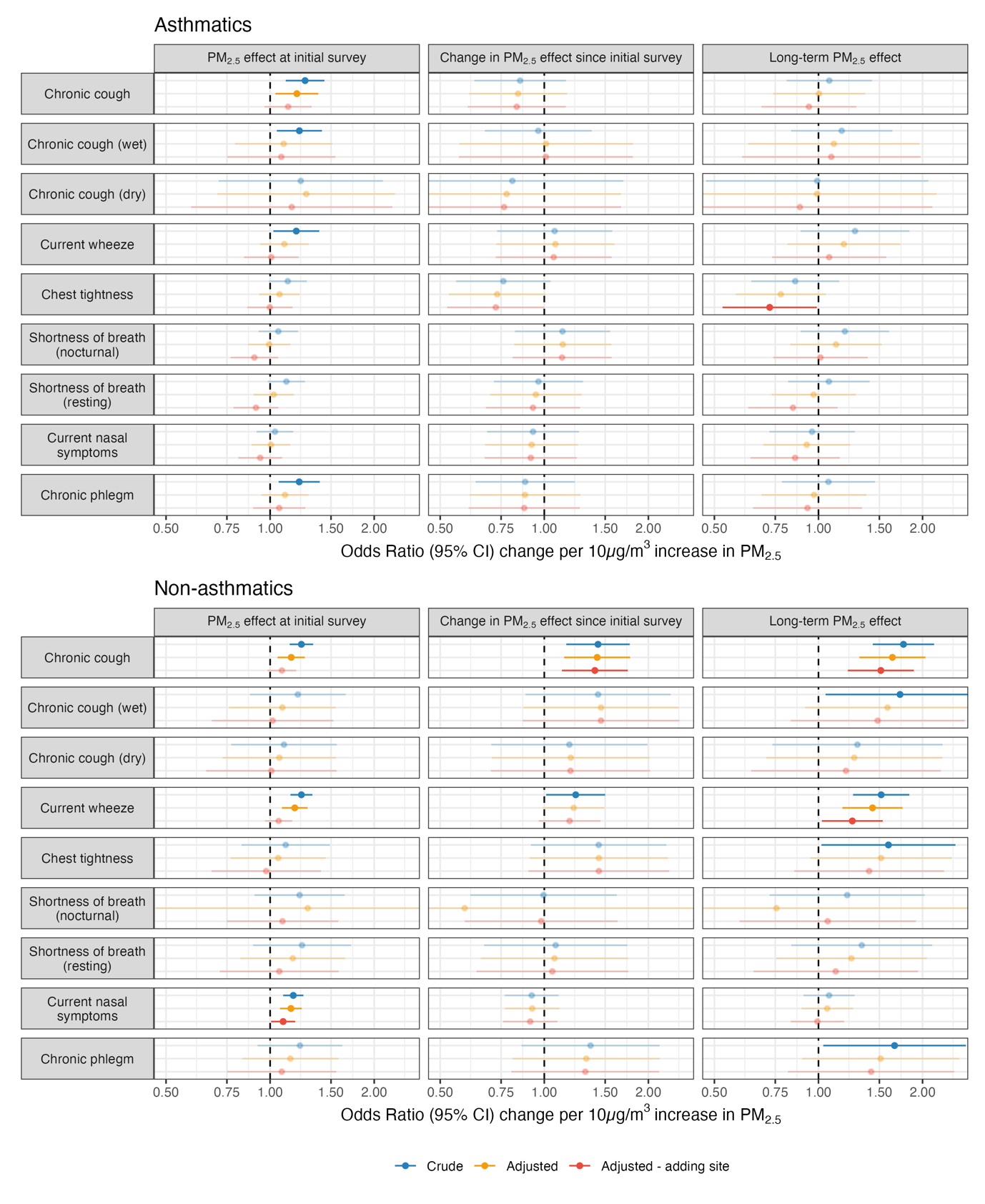


Figure D. Analyses stratified by self-reported by asthma status on effects of 10µg/m^3^ increase in mean coalmine fire-related PM_2.5_ on odds of respiratory symptom at the initial 2016/17 survey (2-3 years post-fire), change between the two survey rounds (interaction term between fire-related PM_2.5_ and survey round), and the cumulative long-term effect at the 2022 follow-up (8.5-9 years post-fire, estimated using linear combination); faded points and intervals indicate non-significant effects

# Deviations from pre-registered analysis plan

This study was pre-registered on the Open Science Framework on 19 July 2022, the month before the follow-up survey commenced, and updated during the survey with a more specific analysis plan on 22 November 2022 [6].

We initially planned to include terms for PM_2.5_ exposure from the 2019/2020 Black Summer bushfire, although the necessary modelled data were not available as of writing. Additionally, we found there was likely little difference in exposure between Morwell and Sale [7,8]. Research questions were clarified to reflect this change.

Confounder adjustment changed several times and was finalised during peer review after receiving a suggestion to justify our approach using a DAG (see Section 2: Model specifications of this document). We opted to use the simplest model indicated by the DAG, rather than include all that were assessed as non-biasing.

Initially, we intended to test two approaches for tobacco use (smoker status versus pack-years to capture) and select one based on model fit but decided to use both. The square root transformation of pack years was not pre-specified, but applied after observing the extreme right-skewed in the distributions in a prior analysis [8]. While adjustment for age was pre-specified as age group, we opted to use the natural spline to better account for non-linear effects while also retaining the rich information of continuous data rather than recoding into categorical variable. Body Mass Index was excluded as a confounder because it only available for those who participated in the follow-up survey.

# Additional resources

## Statistical packages for cleaning and analysing study data

To give credit to authors of the statistical packages used in this paper, we have listed theme here. All analyses were conducted in *R* [9] in the *RStudio* IDE [10]. Specific packages include: *broom.mixed* [11] to produce tidy results tables; *glmmTMB* [12] for mixed-effect logistic regression modelling; *gtsummary* [13] for descriptive tables and univariate comparisons; *haven* [14] to import data from different statistical package formats; *janitor* [15] to clean column names; *mice* [16] for pooling analyses of multiple imputation datasets; *missRanger* [17] for multiple imputation by random forests; *multcomp* [18] to calculate the linear combination of effects and confidence intervals from interaction terms; *naniar* [19] to analyse missingness, patchwork [20] to combine plots; *readxl* [21] to import excel file data; and *tidyverse* [22] to use the suite of tidyverse tools for importation, manipulation, and visualisation tools. All cleaning and analytical code have been archived on a public repository [23].

## Mapping data and software

To create the map of coalmine fire-related PM_2.5_ distribution in Figure 1, we used geospatial data of national, state/territory, and Statistical Area Boundaries from the Australian Bureau of Statistics (copyright information: <https://www.abs.gov.au/website-privacy-copyright-and-disclaimer#copyright-and-creative-commons>) [24], coalmine boundaries from the Victorian Department of Primary Industries [25] and Department of Energy, Environment and Climate Action’s DataShare platform [26], and modelled PM_2.5_ distribution estimates [27]. The map was created using the following R packages: *rmapshaper* [28] to simplify map boundaries, *sf* [29,30] to read and write shapefiles, and *ggplot2* from *tidyverse* [22] to draw and save maps.

# References

1. Burney P, Jarvis D. European Community Respiratory Health Survey III (ECRHS III). Imperial College London; 2011 May. Available: https://www.ecrhs.org/questionnaires-and-protocols

2. Northstone K, Howarth S, Smith D, Bowring C, Wells N, Timpson NJ. The Avon Longitudinal Study of Parents and Children - a resource for COVID-19 research: questionnaire data capture July 2021 to December 2021, with a focus on long COVID [version 2; peer review: 1 approved, 1 approved with reservations]. Wellcome Open Res. 2020;5. doi:10.12688/wellcomeopenres.16020.2

3. Textor J, Van Der Zander B, Gilthorpe MS, Liśkiewicz M, Ellison GTH. Robust causal inference using directed acyclic graphs: the R package ‘dagitty.’ Int J Epidemiol. 2017;45: 1887–1894. doi:10.1093/ije/dyw341

4. Australian Bureau of Statistics. 2033.0.55.001 - Census of Population and Housing: Socio-Economic Indexes for Areas (SEIFA), Australia, 2016. 2018. Available: https://www.abs.gov.au/ausstats/abs@.nsf/mf/2033.0.55.001

5. Prasad S, Gao CX, Borg B, Broder J, Brown D, Ikin JF, et al. Chronic obstructive pulmonary disease in adults exposed to fine particles from a coal mine fire. Ann Am Thorac Soc. 2022;19: 186–195. doi:10.1513/AnnalsATS.202012-1544OC

6. Lane TJ, Carroll M, McCaffrey TA. Long-term respiratory health in a community affected by mine fire. In: Open Science Framework [Internet]. 19 Jul 2022. Available: https://osf.io/yfgb3/

7. Holt NR, Smith CL, Gao CX, Borg B, Lane TJ, Brown D, et al. Lung function may recover after coal mine fire smoke exposure: a longitudinal cohort study. BMJ Open Respir Res. 2024;11: e002539. doi:10.1136/ bmjresp-2024-002539

8. Lane TJ, Carroll M, Borg BM, McCaffrey TA, Smith CL, Gao CX, et al. Long‐term effects of extreme smoke exposure on COVID ‐19: A cohort study. Respirology. 2024;29: 56–62. doi:10.1111/resp.14591

9. R Core Team. R: A Language and Environment for Statistical Computing. Vienna, Austria: R Foundation for Statistical Computing; 2024. Available: https://www.r-project.org/

10. RStudio Team. RStudio: Integrated Development for R. Boston, MA: RStudio, Inc.; 2024. Available: https://www.rstudio.com

11. Bolker B, Robinson D. broom.mixed: Tidying Methods for Mixed Models. 2020.

12. Brooks M E, Kristensen K, Benthem K J ,van, Magnusson A, Berg C W, Nielsen A, et al. glmmTMB Balances Speed and Flexibility Among Packages for Zero-inflated Generalized Linear Mixed Modeling. R J. 2017;9: 378. doi:10.32614/RJ-2017-066

13. Sjoberg DD, Whiting K, Curry M, Lavery JA, Larmarange J. Reproducible summary tables with the gtsummary package. R J. 2021;13: 570–580. doi:10.32614/RJ-2021-053

14. Wickham H, Miller E, Smith D. haven: Import and Export “SPSS”, “Stata” and “SAS” Files. 2023. Available: https://CRAN.R-project.org/package=haven

15. Firke S. janitor: Simple Tools for Examining and Cleaning Dirty Data. 2021. Available: https://CRAN.R-project.org/package=janitor

16. van Buuren S, Groothuis-Oudshoorn K. “mice”: Multivariate Imputation by Chained Equations in R. J Stat Softw. 2011;45: 1–67. doi:10.18637/jss.v045.i03

17. Mayer M. missRanger: Fast Imputation of Missing Values. 2024. Available: https://CRAN.R-project.org/package=missRanger

18. Hothorn T, Bretz F, Westfall P. Simultaneous inference in general parametric models. Biom J. 2008;50: 346–363. doi:10.1002/bimj.200810425

19. Tierney N, Cook D, McBain M, Fay C. naniar: Data Structures, Summaries, and Visualisations for Missing Data. 2021. Available: https://CRAN.R-project.org/package=naniar

20. Pedersen TL. patchwork: The Composer of Plots. 2022. Available: https://CRAN.R-project.org/package=patchwork

21. Wickham H, Bryan J. readxl: Read Excel Files. 2019. Available: https://cran.r-project.org/package=readxl

22. Wickham H, Averick M, Bryan J, Chang W, McGowan L, François R, et al. Welcome to the Tidyverse. J Open Source Softw. 2019;4: 1686. doi:10.21105/joss.01686

23. Lane TJ. Respiratory symptoms after coalmine fire and pandemic: a longitudinal analysis of the Hazelwood Health Study cohort - analytical code. In: Bridges [Internet]. 2023. Available: https://doi.org/10.26180/22596994.v2

24. Australian Bureau of Statistics. 1270.0.55.006 - Australian Statistical Geography Standard (ASGS): Correspondences, July 2011. 2012. Available: https://www.abs.gov.au/AUSSTATS/abs@.nsf/DetailsPage/1270.0.55.006July 2011

25. Department of Primary Industries. Victorian Coal: A 2006 Inventory of Resources. Morwell, Victoria: GHD Pty Ltd; 2007 Aug p. 59. Report No.: 31/20049/7979. Available: http://earthresources.efirst.com.au/product.asp?pID=645&cID=6&c=71757

26. Department of Energy, Environment and Climate Action. Victorian Coal Fields. Melbourne: DataShare; 2007. Available: https://datashare.maps.vic.gov.au

27. Luhar AK, Emmerson KM, Reisen F, Williamson GJ, Cope ME. Modelling smoke distribution in the vicinity of a large and prolonged fire from an open-cut coal mine. Atmos Environ. 2020;229: 117471. doi:10.1016/j.atmosenv.2020.117471

28. Teucher A, Russell K. rmapshaper: Client for “mapshaper” for “Geospatial” Operations. 2023. Available: https://CRAN.R-project.org/package=rmapshaper

29. Pebesma E. Simple Features for R: Standardized Support for Spatial Vector Data. R J. 2018;10: 439–446. doi:10.32614/RJ-2018-009

30. Pebesma E, Bivand R. Spatial Data Science: With Applications in R. New York: Chapman and Hall/CRC; 2023. Available: https://doi.org/10.1201/9780429459016
