# Supplementary material for: Respiratory symptoms after coalmine fire and pandemic: A longitudinal analysis of the Hazelwood Health Study adult cohort
Source: PLOS Glob Public Health. 2025 Jan 22;5(1):e0004186. doi: 10.1371/journal.pgph.0004186 (PMC11753711; doi:10.1371/journal.pgph.0004186)
Supplement: Attachment — Submitted filename: Reviewers comments.DOCX [file pgph.0004186.s002.DOCX]

**Reviewer’s Comments.**

1. Ensure the consistent use of $PM_{2.5}$ in your manuscript. While some sections in the article had PM 2.5, some were written as $PM_{2.5}$.
2. **Line 11:** Rewrite as “Over the years, research has identified numerous associations between $PM_{2.5}$ exposure and deteriorating respiratory health”
3. **Line 13:** What is the full meaning of GP?
4. **Line 17:** Four years instead of “4 years”
5. **Line 21**: In late 2019, a new and significant threat to respiratory health emerged with the onset of the COVID-19 pandemic**.**
6. **Line 23:** $PM_{2.5}$ exposures may even increase the likelihood that SARS- CoV-2 infection leads to a cytokine storm. Remove the word “even”.
7. The longitudinal study provides evidences of the effect of extreme exposure to $PM_{2.5}$ on the respiratory. The sample size isn’t large enough to capture the effect of $PM_{2.5}$ exposure, although this has been addressed as one of the limitation of the study. The method in the abstract did not address the method of analysis used in analyzing the data.
