# Supplementary material for: Respiratory symptoms after coalmine fire and pandemic: A longitudinal analysis of the Hazelwood Health Study adult cohort
Source: PLOS Glob Public Health. 2025 Jan 22;5(1):e0004186. doi: 10.1371/journal.pgph.0004186 (PMC11753711; doi:10.1371/journal.pgph.0004186)
Supplement: Attachment — Submitted filename: Response to reviewers .docx [file pgph.0004186.s003.docx]

Dear Professor Babu

**RE: PGPH-D-24-00948 *Respiratory symptoms after coalmine fire and pandemic: a longitudinal analysis of the Hazelwood Health Study adult cohort***

On behalf of the research team, I would like to thank you and the reviewers for your valuable comments. The manuscript has been updated in accordance with these suggestions and where the suggestions were not incorporated, we have provided a detailed explanation.

In addition, we have made two updates to our analytical approach that were unprompted by this review. The first was modifying the nasal allergy variable; we had mistakenly used a variable that referred to nasal allergies that preceded the mine fire. This has now been updated to nasal symptoms in the 12 months preceding interview (please refer to 1.1 in the supplementary materials). There is now one instance where fire-related PM_2.5_ was associated with greater prevalence of current nasal symptoms.

The second update was swapping out multiple imputation by chained equations (MICE) for random forest multiple imputation. This approach was considerably faster and has some methodological advantages over chained equations. We identified no meaningful difference in results.

**Editor**

**Editorial Comment 1:**

The authors need to explain how the measurement error in exposure assessment during the coalmine fire was minimized by combining self-reported time-location diaries with modelled PM2.5 estimates. Specifically, the spatial models to estimate individual PM2.5 exposure during the coalmine fire can have potential ecological bias. They can result in exposure misclassification due to variations in individual movement and duration of stay in the assessed areas.

**Author response:**

In this study, we combined time-location diaries and spatial and temporal modelling of PM_2.5_ from the Hazelwood coalmine fire to generate individual-level estimates of PM_2.5_ exposure. While this did not eliminate ecological bias entirely (i.e., at best, the models have 100m^2^ resolution, which assumes heterogeneity across the entire area), we nevertheless believe it was largely mitigated by allowing exposure to vary based on time and the participant’s movements. In addition, the spatial resolution was quite high, reaching 100m^2^ in areas around the mine.

To clarify this point, we have added a line to the methods (2.1.1), which specifies the advantage of this approach: “This strategy [combining the diaries and modelled PM_2.5_ data] accounted for individual-level differences in fire-related PM_2.5_ exposure due to participant movement throughout the mine fire period and variations in smoke distribution over space and time.”

Further, we have updated the strengths and limitations (4.1) to specifically refer to issues around misclassification due to participant recall and limits in the accuracy of modelled PM_2.5_ data.

**Editorial Comment 2:**

Further, it is not clear if the outcome was assessed and excluded at baseline to follow-up only those at risk of developing respiratory symptoms. Also, the frequency of administering the question (whether weekly, monthly, or only towards the end) is not provided. It is unclear how the authors excluded prevalent symptoms at baseline. Since respiratory symptoms can be associated with other exposures, authors need to specify how the specificity and temporality are established between PM2.5 and the outcome.

**Author response:**

With the addition of a DAG (as suggested below), we have clarified our analytical approach. Our language was confusing, using “baseline” and “initial” survey interchangeably. There is only an initial survey, conducted 2-3 years post-fire; we have now removed references to “baseline” from the manuscript.

In longitudinal analyses, we did not exclude baseline respiratory symptoms (i.e., those reported at initial survey) as they are included in the model as outcome variables. In the cross-sectional analysis investigating potential moderating effects of COVID-19 at the follow-up survey, we have modified the analysis to no longer adjust for baseline survey respiratory symptoms (i.e., those reported at initial survey). As we now understand it, adjusting for baseline symptoms in the PM_2.5_ * COVID-19 interaction model was actually adjusting for a mediator, as PM_2.5_ exposure (from 2014 mine fire) affected prevalence of symptoms at baseline (recorded in 2016/17 survey), biasing PM_2.5_ effects towards null in this analysis.

As for the frequency of the survey, each was administered only one time to each cohort member. Part of the confusion was likely our interchangeable use of the words “initial” and “baseline” survey; there was only an initial and follow-up survey. We have clarified the text in the methods section (2.1, 2.2).

**Editorial Comment 3:**

The authors need to specify the hypothesis using a directed acyclic graph and justify the inclusion of confounders. For example, it is unclear how being fully vaccinated against COVID-19 can be a confounder given that it is not associated with PM 2.5 exposure.

**Author response:**

We have added DAGs to better justify our models, which can be found in Section 2 of the supplementary materials. As suggested, the DAG indicated that adjusting for COVID-19 as a confounder of PM_2.5_ exposure would introduce bias. There were also some other changes to the model specifications, which we detail in the methods (2.2). However, the findings remained largely consistent.

**Editorial Comment 4:**

Only 25% of the original cohort's 4,056 members participated in the follow-up survey. The authors need to explain why the differential loss to follow-up might have biased the study results. The discussion lacks details on potential threats to internal validity, such as differential loss to follow-up, measurement error, and the selection of confounders.

**Author response:**

To make the effects of attrition more transparent, we have added a new section (3.1) and Table S2 to the supplemental materials, which cover how those who participated in the follow-up differed from those who did not. In the revised manuscript, we now note this in the results (3.1). And in the discussion of strengths and limitations (4.1), we have added some consideration of selection bias due to loss-to-follow-up, and how this was partially accounted for by statistically adjusting for these factors.

**Reviewer 1**

**Reviewer comment 1:**

It was an interesting paper on the long term impact of PM2.5 exposure on respiratory health.

Here are my specific comments

2. Methods

2.1.1

Exposures

Figure 1: What were the PM2.5 levels over the years (post 2014)?

**Author response:**

In a separate paper (1), we reported time series data on annual PM_2.5_ levels in Morwell, the rest of the Latrobe Valley, Sale, and Victoria. The graph has been reproduced below It was fairly similar and stable across our study sites (Morwell and Sale), between about 7-8 µg/m^3^, with the exception of the “Black Summer” period, when both sites saw similar peaks.

We have updated the methods section on exposures (2.1.1) with the following line: “There was little evidence that background ambient PM_2.5_ differed between these areas [Morwell & Sale] (1).”

**Reviewer comment 2:**

2.2 Statistical analysis

Page No. 7

Each model adjusted for the presence of the specific respiratory symptom at the baseline survey (e.g., the model assessing whether COVID-19 exacerbated effects of PM2.5 exposure on prevalence of chronic cough at the 2022 follow-up survey adjusted for presence of chronic cough at the 2016/2017 baseline survey).

I think the word ‘was’ is missing in the sentence.

Each model was adjusted for the presence of the specific respiratory symptom at the baseline survey (e.g., the model assessing whether COVID-19 exacerbated effects of PM2.5 exposure on prevalence of chronic cough at the 2022 follow-up survey, was adjusted for presence of chronic cough at the 2016/2017 baseline survey).

**Author response:**

Thanks for catching that. However, as you will see in our response to the editor, we have added a DAG and modified our models. One consequence is that we removed baseline/initial survey respiratory symptoms as a confounder, as this would be along the causal pathway from PM_2.5_ -> symptoms at follow-up, making symptom at initial survey a mediator (baseline symptom has been removed from the DAG as it is now obvious to us that it is a mediator and should not be included in the model, and the need to simplify the representation of our causal assumptions). The entire paragraph has been removed as it is now incorrect.

**Reviewer comment 3:**

3. Results

3.1 Descriptives

Page 9

National Death Index data (31), which were linked to cohort data after the survey was completed, indicated 143 invited cohort members (7.7%) died prior to the close of the survey window (December 2022)

Correct the percentage. It is 5.8% (143/2458)

**Author response:**

Thanks for catching that. We had used non-participants as the denominator (1846), but you are correct that we should have used the invited cohort.

**Reviewer comment 4:**

4. Discussion

Could include comparison with studies on long term impact of wildfires on respiratory outcomes since there is a mention of the 2019/2020 Black Summer bushfires.

**Author response:**

While we agree this would provide valuable context, we were unable to identify high-quality research that investigated effects of wildfire on chronic cough and other respiratory symptoms. The best two deal with respiratory symptoms two years after a fire in Lake Seeley (2) or respiratory symptoms in monkeys a year after a fire (3). Neither seems to be of much relevance long-term respiratory outcomes.

**Reviewer comment 5:**

4. Discussion

Increase in current wheeze was isolated to non-asthmatics, and there was a reduction in chest tightness among asthmatics. This is an interesting finding, could include supporting literature.

**Author response:**

We have added a line and a reference to Gan et al. 2020 (4), who found an increase in the use of asthma rescue medicine (SABAs) after the 2013 Oregon wildfire season. While there were other papers, they mainly focused on children or we otherwise found the quality of the evidence questionable.

**Reviewer 2**

The study is highly relevant as it allows one to analyze the long-term effects of extreme “point-in-time” exposure to PM2.5. Such studies are not common, particularly when there is the possibility of using historical data to analyze long-term effects. The study also highlights the limited analysis of the potential cumulative effects between SARS-CoV-2 infection and respiratory symptoms. Specific suggestions for revision could enhance the quality of the study.

**Reviewer comment 1:**

Abstract
The abstract does not clearly state the study's objective. I suggest including it, considering the research questions presented at the end of the introduction.

**Author response:**

Good point. This has now been updated in the “Objectives” section of the abstract.

**Reviewer comment 2:**

Abstract
In the methods section, I suggest distinguishing between the models used to evaluate the long-term effects of PM2.5 exposure and the association of these effects with SARS-CoV-2 infection.

**Author response:**

Done.

**Reviewer comment 3:**

Introduction
-2nd Paragraph – In this second paragraph, what does GP mean?

**Author response:**

GP: General Practitioner, usually the first point of primary care for most health concerns in Australia, the UK, and other commonwealth nations. We have now spelled out the acronym in the manuscript.

**Reviewer comment 4:**

Introduction
- 2nd Paragraph – At the end of the paragraph, an increase in outcomes associated with the 2014 coalmine fire is described. Does this increase refer to other locations or other periods analyzed?

**Author response:**

This depends on which study is being referenced. Some referred to the adult cohort, in which case these were the same participants whom we analysed in this study, just with additional follow-up data. Those referring to respiratory function rely on a specific subset of the adult cohort, the respiratory stream. Others used anonymous extracts of health data from hospitals and ambulance services, which covered a much wider area – sometimes the entirety of regional Victoria – and covered different time periods.

Our concern was that all these different sources were confusing, and that any attempt to clarify the origins of each study (beyond the citation) would only reduce clarity. Therefore, rather than clog the introduction with repetition of sources, we have tried our best to convey an overarching summary.

**Reviewer comment 5:**

Methods
- Survey – It is not clear whether the profile of survey respondents changed compared to the 2016/2017 survey. It also does not mention how the survey was conducted, whether through personal interviews or using tools such as mobile apps. I suggest including this information.

**Author response:**

Details on how follow-up participants differed from other members of the cohort have now been provided in the results (3.1) and supplementary materials (1.1, Table S2), as well as discussion in the strengths and limitations (4.1).

We have now provided new information about how the surveys were conducted in the participants section of the methods (2.1).

**Reviewer comment 6:**

Methods
- Exposure – I suggest including an explanation for choosing Sale as a control.

**Author response:**

We have added more text to the section on participants (2.1), which emphasises that Sale was both near to Morwell (~60km) and the mine but unexposed to smoke from the fire. We also add more detail that they were residing in selected areas of Sale that better matched Morwell in socioeconomic terms.

**Reviewer comment 7:**
Methods
- Statistical Analyses – Regarding missing data, did this refer to the absence of some responses or respondents?

**Author response:**

This refers to missing responses or cells, either because a participant refused or couldn’t provide an answer; e.g., in Table S1, we reported that *n* = 5 people in the original survey did not report an age or date of birth, *n* = 44 did not provide smoking status, etc.

We have added a note to the reader to refer to Table S1 for information on missing data.

**Reviewer comment 8:**

Results
- Regarding missing data, it is unclear how many imputations were made relative to the data obtained.

**Author response:**

We have updated the section on statistical analysis (2.2) to clarify how we arrived at the number of imputations, which was equivalent to the proportion of cases with missing data, and the results (3.1) to note that 8.5% of cases had missing data, therefore we conducted 9 imputations. In addition, we have modified our approach from multiple imputation by chained equations (MICE) to random forest multiple imputation.

**Reviewer comment 9:**

Results
- To better understand the results, I strongly suggest including p-values in the text when significance is obtained and, if possible, the final models for each analysis in addition to Figures 2 and 3.

**Author response:**

While we understand the reviewer’s position, we think there are several reasons not to provide p-values and final models in the text.

1. Reporting models and p-values for each analysis would clutter up the text considerably, as we have three results for each outcome (crude, adjusted, adjusted with site) We think reporting p-values for just the final model would still add clutter, reducing our paper’s readability.
2. While we used p-values in our plots to highlight significant values in the figures (i.e., significant figures are bold), we think reporting them in text would put too much emphasis on the significance dichotomy. We think using these forest plots to convey point values, precision (in terms of confidence intervals), and proximity to the non-significance threshold in one figure provides more meaningful information.
3. Regardless, p-values for each model have been provided in Tables S3 and S4 in the supplementary materials.

**Reviewer comment 10:**

Discussion
- 1st Paragraph – At the end of the paragraph, it is mentioned that other locations worldwide also experienced an increase in chronic cough following exposure to extreme smoke events. I suggest referencing these other events and their consequences in exacerbating chronic cough.

**Author response:**

We have updated the text to clarify our intent, which was not to draw attention to specific events and evidence for increases in chronic cough observed elsewhere, but rather: 1) highlight that increasing prevalence of chronic cough was bad for our cohort, and 2) the implications are concerning in a world where exposure to smoke from landscape fires is increasing due to climate change. We hope this modification is satisfactory.

Further, as we note to Reviewer 1, we were unable to identify much evidence on long-term respiratory symptoms and wildfire that focused on adults and was of acceptable quality.

**Reviewer comment 11:**

Discussion
- 2nd Paragraph - The 2019/2020 Black Summer bushfires are mentioned, but there is no further explanation of what this event was. Considering the publication's global reach, I suggest explaining what this refers to and including references, including regarding the prevalence of chronic cough.

**Author response:**

We have now provided detail in the Discussion as to what the “Black Summer” was, and more clearly described what we think could have happened with this later smoke event (Black Summer) exacerbating the effects of this earlier smoke event (Hazelwood coalmine fire).

**Reviewer comment 12:**

Discussion
- Finally, there is no mention of hospitalization data or records of care related to the respiratory symptoms analyzed. Although such data were not used in the proposed models, I suggest including them in the discussion or at least indicating the rationale for not using this data.

**Author response:**

We have expanded the introduction to refer more to evidence from the Hazelwood Health Study’s analyses of hospital and ambulance data, which we believe is necessary to set up the later section we have added to the discussion. This focuses largely on why results may not be consistent across studies, primarily that health service use is not a direct measure of health need, but also access.

**Reviewer 3**

**Reviewer comment 1:**

The longitudinal study provided the necessary data sufficient to address the research questions. The longitudinal study provides evidences of the effect of extreme exposure to PM_2.5 on the respiratory. The sample size isn’t large enough to capture the effect of PM_2.5 exposure, although this has been addressed as one of the limitation of the study. The method in the abstract did not address the method of analysis used in analyzing the data.

**Author response:**

We have updated the methods (2.2) to clarify what analytical approach we used. Otherwise, as the reviewer notes, we report issues around statistical power due to a small sample in the strengths and limitations section (4.1).

# Additional references

1.	Lane TJ, Carroll M, Borg BM, McCaffrey TA, Smith CL, Gao CX, et al. Long‐term effects of extreme smoke exposure on COVID ‐19: A cohort study. Respirol. 2024 Jan;29(1):56–62.

2.	Orr A, A. L. Migliaccio C, Buford M, Ballou S, Migliaccio CT. Sustained Effects on Lung Function in Community Members Following Exposure to Hazardous PM2.5 Levels from Wildfire Smoke. Toxics. 2020 Aug 5;8(3):53.

3.	Black C, Gerriets JE, Fontaine JH, Harper RW, Kenyon NJ, Tablin F, et al. Early life wildfire smoke exposure is associated with immune dysregulation and lung function decrements in adolescence. Am J Respir Cell Mol Biol. 2017;56(5):657–66.

4.	Gan RW, Liu J, Ford B, O’Dell K, Vaidyanathan A, Wilson A, et al. The association between wildfire smoke exposure and asthma-specific medical care utilization in Oregon during the 2013 wildfire season. J Expo Sci Environ Epidemiol. 2020 Jul;30(4):618–28.
